# Boosting the Immune System, From Science to Myth: Analysis the Infosphere With Google

**DOI:** 10.3389/fmed.2019.00165

**Published:** 2019-07-25

**Authors:** Arthur Cassa Macedo, André Oliveira Vilela de Faria, Pietro Ghezzi

**Affiliations:** ^1^Faculdade de Medicina, Universidade Federal de Minas Gerais, Belo Horizonte, Brazil; ^2^Brighton & Sussex Medical School, Brighton, United Kingdom

**Keywords:** internet, immunity, vaccines, antioxidants, google, vitamins, complementary and alternative medicine

## Abstract

**Background:** The concept that one can “boost” immunity is a popular one. Although the only evidence-based approach to this is vaccination, the lay public is exposed to a wide range of information on how to boost immunity. The aim of this study was to analyze such information available on the Internet.

**Methods and findings:** We visited 185 webpages returned from a Google search on “boost immunity” and classified them by typology (blogs, commercial, government, no-profit, news, professional, scientific journals) and by using standard indicators of health information quality (JAMA score, HONCode). We then analyzed their content in terms of disease and “boosters” mentioned. Commercial and news websites represented one third of the results each. Of the 37 approaches to boost immunity recorded, the top ones were diet (77% of webpages), fruit (69%), vitamins (67%), antioxidants (52%), probiotics (51%), minerals (50%), and vitamin C (49%). Interestingly, vaccines ranked 27th, with only 12% of webpages mentioning them.

**Conclusions:** Commercial websites are an important component of the information available to the public on the topic, and thus contribute providing biased information.

Immunity is the main mechanism of host defense against infectious agents, demonstrated by the enormous success of vaccination in eradicating disease (1). The concept that vaccines are the most effective means of preventing infection is well-recognized, both by public health authorities and by the public. Daniel Davis wrote, “the public are fascinated by the connections between lifestyle choices and immunity because there may be practical implications for what it takes to be healthy” (2). There is, in particular, a public interest on increasing immune defense easily. Probably, the most popular belief is about the use of vitamin C to prevent infection, an idea that became popular after a series of scientific articles by Linus Pauling, who advocated the intake of larger amounts than those recommended at the time (3, 4).

The popularity, among lay persons, of this concept is such that “Improving the body's immune system” is the top reason for consuming nutritional supplements (5, 6). The “immune boosters” market includes vitamins, minerals, antioxidants, probiotics, and “functional foods” as well as other complementary and alternative medicine (CAM) approaches. A study based on the US National Health and Nutrition Examination Survey showed that over 50% of the US population reported the use of supplements (7). This has a huge commercial impact, with the global market of dietary supplements estimated around 133 billion USD (8).

An additional issue is whether the use of non-evidence based approaches to boost immunity can be considered an effective alternative to vaccination. This is a particularly important aspect at a time when vaccine hesitancy is becoming a major threat to global health, as indicated by the World Health Organization (9). A study on 9,000 US children has shown that exposure to some complementary therapies, including chiropractice and other types of alternative medicine (excluding multivitamins/multi-minerals) is associated with a lower uptake if influenza vaccine (10), although this was not observed in adults (11). Similar results were obtained in a survey of over 9,000 Australian women, with those using naturopathy or herbal medicine less likely to vaccinate against influenza (12). It is important to note that, in two of the cited studies, use of multivitamins/multiminerals was associated with a higher vaccination rate (10, 12). However, when vaccine confidence was studied in a survey on attitudes to vaccination among 1,250 Australian adults, use of most CAM, including vitamins, was associated with lower levels of vaccination endorsement (13). It is therefore important to establish what type of information the lay public is exposed to as this is likely to be the basis of their knowledge of the topic and have important consequences on public health.

The present study aimed at obtaining a picture of the information the public is exposed to on this topic. In recent years, it became clear that we live in an online informational environment that Floridi defined as “infosphere” (14). Because most of the sources of information, including books, news outlets, governmental, and professional organizations, are now available online, we used the Google® search engine to obtain a sample of the information available on the topic, using a methodology we have successfully applied to analyze knowledge about other health-related topics (15–17).

The webpages returned were then analyzed in terms of type of website, whether, for instance, from commercial entities, news outlets, etc. We also analyzed the webpages returned by the search in terms of standard indicators of health information quality (HIQ). The first is the Journal of the American Medical Association (JAMA) score, a widely used HIQ tool where the transparency/trustworthiness of a webpage is assessed for the presence or absence of the following information: author, date, external references, and ownership of the website (18). We also looked for the presence of the Health On the Net (HON) code certification provided by an independent organization, the HON Foundation, based on a code of conduct comprising several criteria of quality and transparency (19).

## Methods

### Data Collection and Classification

We searched “boost immunity” in Google.com from Brighton, UK in November 2018. The search was done using the browser Google Chrome®, deleting cookies and browser history to limit personalization of the search results returned, although geolocalization could not be prevented as this is linked to the IP address used for the connection to the Internet. The choice of Google® as a search engine was because it has over 90% of the search engine market share (20).

We then transferred the first 200 Uniform Resource Locators (URLs) in the search engine result page (SERP) to a spreadsheet. To allow some margin in the transfer of the hyperlink we downloaded the first 204 results. Using a workflow described previously (15–17), we then visited each webpage and read the content. Nineteen pages were excluded because they were clearly irrelevant, not accessible (dead links) or required registration or paywall to access them. The remaining 185 webpages were then analyzed in terms of typology of website, HIQ indicators and content.

Websites were first classified according to their typology, as described in Table 1. While all other classifications we made were based on the presence of a particular text, assigning a typology may be subjective. Therefore, this was done by two raters and disagreements were discussed until we agreed on a classification. We had a 77% agreement on the classification by typology. The inter-rater agreement gave a Cohen's kappa coefficient of 0.721 with a standard error of 0.035, and a 95% confidence interval, from 0.652 to 0.790. This strength of agreement is considered: “good.” The weighted Kappa was 0.809, considered ‘very good.’ It should be noted, however, that the agreement differed for the typologies and was as follows: blogs, 75%; commercial, 95%; excluded, 90%; government, 100%; health portals, 22%; news, 88%; no profit, 100%; professional, 100%; scientific journals, 75%. This means that the agreement was very good for all typologies but very low for health portals, suggesting that any conclusions made on the latter typology would be very weak.

**Table 1:** Examples of websites typologies.

**Typology**	**Examples**
Blog	https://foodrevolution.org/blog/how-to-boost-immune-system/
	https://www.lorizanini.com/boostyourimmunity/
Commercial	https://draxe.com
	https://www.humnutrition.com
Government	https://nccih.nih.gov
	https://www.nasa.gov
Health news	https://www.womenshealthmag.com
	http://time.com
	https://www.theguardian.com
Health portal	https://www.webmd.com
	https://www.medicinenet.com
Non-profit organization	https://nutritionfacts.org
Professionals	https://my.clevelandclinic.org
	https://www.health.harvard.edu
Scientific journals	https://www.sciencedirect.com
	https://www.scientificamerican.com
Other	https://en.wikipedia.org
	https://blog.paleohacks.com

### Indicators of Health Information Quality

Each webpage was then evaluated for standard HIQ indicators. The JAMA score was calculated assessing the presence or absence of the following information: author, date, external references, and ownership of the website (18). For each of these we assigned a score of 1, so that the total JAMA score ranged from 0 to 4. We also looked for the presence of the HONcode certification on the page (19). When deciding the presence or absence of information we applied the three-click rule, which requires the information to be present, no more than three clicks away from the webpage under analysis (21).

### Content Analysis

A content analysis was then performed, recording the disease mentioned in the webpage, if any, and the type of approach mentioned in the context of boosting immunity. The list was compiled as long as the webpages were read and included supplements, dietary, and lifestyle recommendation, complementary approaches such as chiropractic or yoga, as well as medically-approved approaches such as hygiene or vaccination. Because webpages mentioning, for instance, vitamin C, could mention either eating fresh fruit or buying a vitamin C supplement or some webpages may state you should eat fruit and not supplements, we recorded the stance about supplements, whether negative or positive. Finally, we recorded whether any webpage had a negative stance about vaccines, either anti-vaccine or just vaccine-skeptical.

In some cases, we analyzed the usage of the word “supplement” or “University” in a specific set of webpages. This was done using corpus analysis. Briefly, text corpora were extracted from the webpages to be analyzed using WebBootCaT, an online tool for bootstrapping text corpora from the Internet. Then the concordance of specific words were obtained using the corpus analysis software Sketch Engine by Lexical Computing, Brno-Královo Pole, Czechia (22). Results are shown as “Key Word In Context,” i.e. a list of all the occurrences of the search word in the webpages with an equal amount of context on the left and on the right.

The spreadsheet with the raw data is available as Supplementary File 1 to allow re-analysis by different methods.

### Statistical Analysis

Statistical analysis was performed with GraphPad Prism 7.04 for Windows using the tests described in the text. The number of symptoms mentioned in a specific typology of websites was compared with the rest of the search, rather than with the whole search, to avoid comparing overlapping data. To compare two groups, non-parametric tests were used: a two-tailed Mann-Whitney test for comparing two groups and a two-tailed Kruskal–Wallis test in case(s) of multiple comparisons. Frequency of typologies of websites in the first 10 results in the Google search were compared with that in the rest of the search using a two-tailed Fisher's exact test. The level of significance was set at *P* < 0.05. When a Fisher's test was performed in multiple comparisons, this was followed by a Benjamini–Hochberg correction set at a false discovery rate of 5%.

Ethical approval was not sought because this research did not involve human participants.

## Results

### Typology of the Webpages Returned in the SERP

This first analysis looked at the composition of the SERP (185 webpages) and the top 10 webpages returned by Google in terms of typology of websites, which is essentially the details of the public or private organization who owns them. The results in Table 2 show that the two main typologies returned by Google were “commercial” and “news.” However, commercial websites were given a low visibility by Google, at least in this search, and none of them were present in the top 10 results. On the contrary, news websites ranked high, as their frequency in the top 10 was twice that in the total search. The difference in the frequency of commercial and professional websites in the top 10 webpages compared to the rest of the SERP (e.g., those in positions 11–204) was statistically significantly (*P* = 0.032 by a two-tailed Fisher test for commercial websites and *P* = 0.014 for professional websites), while the one for news websites was not. Thus, Google gives a significantly higher visibility to professional websites wile ranking commercial websites significantly lower.

**Table 2:** Type of websites in the SERP.

**Typology**	**Whole search**	**Top 10**
Commercial	60 (32%)	–
News	59 (32%)	6
Blog	18 (10%)	–
Professional	16 (9%)	4
Health portal	10 (5%)	–
No-profit	9 (5%)	–
Other	7 (4%)	–
Government	3 (2%)	–
Scientific journals	3 (2%)	–

*Percent of websites in each typology is shown in parenthesis (no. of webpages in the whole search, 185; in the top 10, 10)*.

### Standard Indicators of Health Information Quality: JAMA Score and HONcode

**Figure 1:**
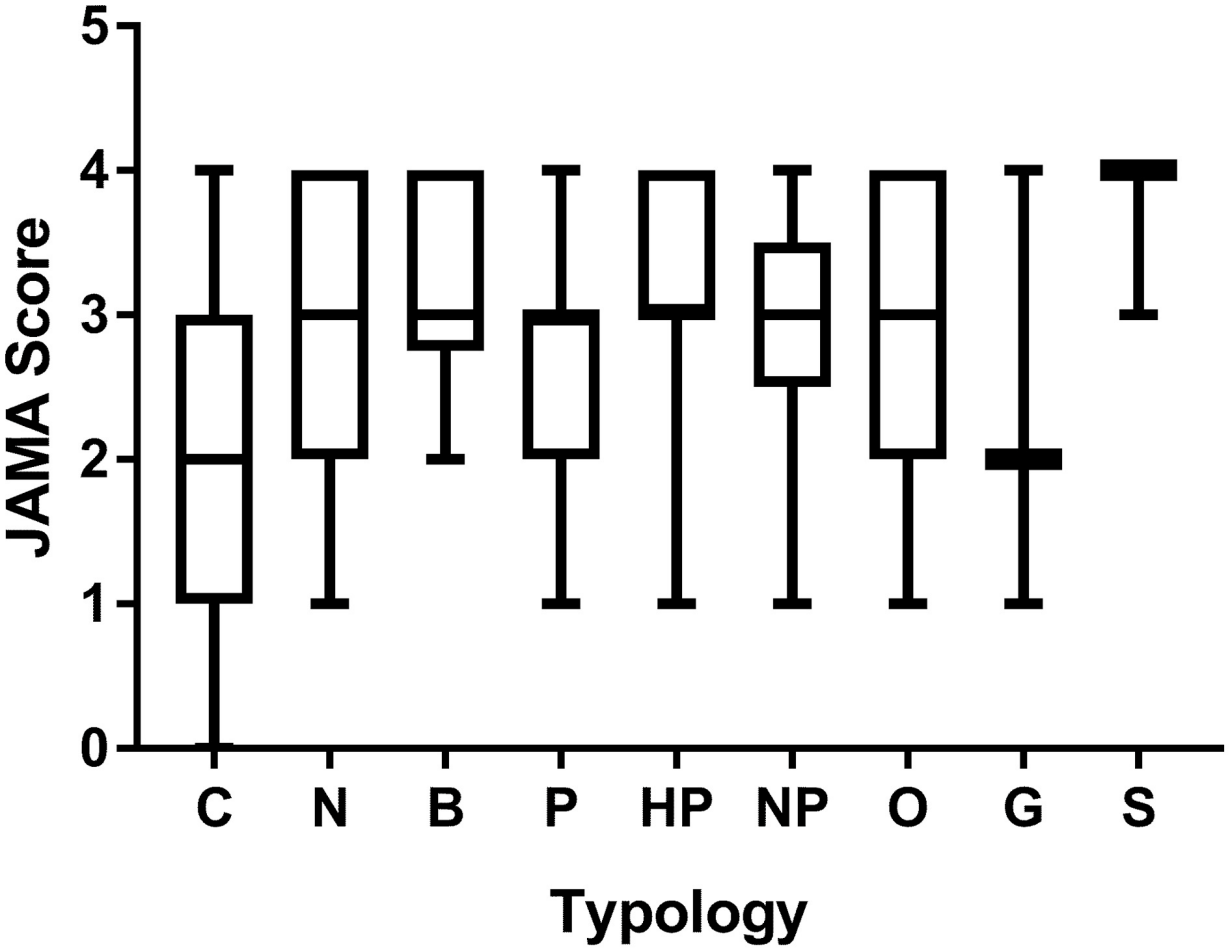
JAMA score of webpages of different typologies. Data are median, IQR, minimum, maximum. Number of webpages for each group was: commercial, 60; News, 59; Blog, 18; Professionals, 16; Health portal, 10; Non-profit organization, 9; Other, 7; Government, 3; Scientific journals, 3.

We then analyzed each webpage for the indication of authorship, date, ownership of the webpage, and presence of external references (the JAMA score, ranging from 0 to 4 depending on how many of these criteria were met). Figure 1 reports the JAMA score for each typology of websites. Commercial webpages scored the lowest (mean 2.0; median 2, [IQR:1, 3]; *n* = 60) which was significantly lower than news, blogs, and health portals (respectively, by *P* < 0.0001, *P* < 0.01, and *P* < 0.05 using ANOVA followed by Tukey's multiple comparison test). The JAMA score of commercial webpages was also significantly lower when compared with the rest of the SERP (all other typologies, median 3, [IQR:2, 4]; *n* = 125) with *P* < 0.0001 by a two-tailed Mann-Whitney test). Only nine webpages displayed a HONcode certification, of which five were news, four health portals, and one a no-profit. Despite being the largest typology in the SERP, none of the commercial websites displayed a HONcode certification. The conclusion from this analysis is that the information quality of commercial websites, as assessed by two tools that assess webpages' transparency and trustworthiness is lower than in other typologies of websites.

### Content Analysis

As the tools to measure HIQ described above do not take into account the scientific content of the webpage, we then visited each webpage looking at which disease conditions and approach to boost immunity they mentioned. Table 3 lists the most frequent conditions or disease processes mentioned in the webpages. We specifically recorded mention of respiratory disease, despite these largely overlap with infectious disease, mainly because of the popularity of the idea that vitamins can prevent influenza and the common cold. Infectious and respiratory disease were the most frequently mentioned in the 185 webpages.

**Table 3:** Disease conditions mentioned in the SERP.

**Condition**	**No. webpages**	**%**
Infections	153	83
Respiratory	111	60
Inflammation	61	33
Cancer	53	29
Gastrointestinal	31	17
Chronic diseases	28	15
AIDS	11	6

*Total no. of webpages, 185*.

**Figure 2:**
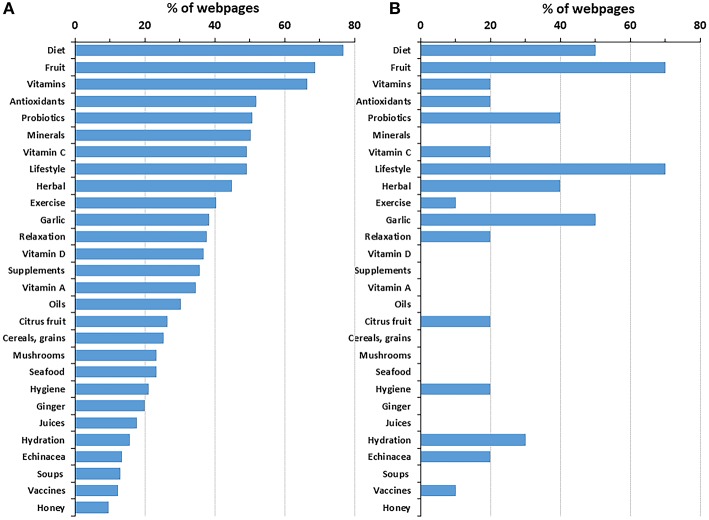
Content analysis of the whole SERP **(A)** and the top-10 webpages **(B)**. Data show the percentage of webpages (**A**, *n* = 185; **B**, *n* = 10) mentioning a specific approach in the context of boosting immunity. Only the approaches that were mentioned in at least 10% of total webpages are shown.

The frequency of the various approaches mentioned (with a positive or neutral stance) to “boost immunity” is shown in Figure 2, panel A (for the whole search) and B (for the top 10 webpages). In the whole search, a healthy diet and fruit/vegetables came on top, along with vitamins. Antioxidants, probiotics, minerals, and vitamin C were also frequently mentioned, in half of the webpages or more, however, most of them referred to diet. Supplements (including vitamins) were mentioned in 36% of the webpages. A number of other approaches were mentioned by a small percentage of webpages (<10%), including chocolate, avoiding excess of medications, breastfeeding, cryotherapy, chiropractic, phytoncides, active sexual life, controlling allergies, and sauna (not shown).

Interestingly, just 23 webpages (12%) mentioned vaccines (also “jab” or “shot” in British and American English, respectively) as a way of boosting immunity. Among these, there were 11 mentions of vaccines in general, 16 of influenza vaccine, two of hepatitis A, two of pneumococcus, two of shingles, one of meningococcus, and one of tetanus-diphtheria-acelluar pertussis vaccine. The prevalence of the influenza vaccine over other types of vaccines is in agreement with the focus on respiratory disease in the webpages, as described earlier (Table 3).

Although the analysis of 185 webpages represents a sample of the information available online, the ranking provided by the search engine is key, as the first results presented to the viewer have a higher chance to be read. In Figure 2, a comparison between panels A (all 185 webpages) and B (top 10 pages) highlight whether webpages mentioning specific interventions are ranked higher by Google. Diet and fruit, the two most frequently mentioned interventions are equally present in the top 10 pages. Vitamins (including vitamins in general, vitamins C, D, and A), antioxidants, oils, and minerals were mentioned less frequently or not mentioned at all (vitamins D and A, oils, minerals) in the top 10 result pages. This might reflect the fact that none of the 60 commercial webpages were present in the top 10 pages.

We also recorded whether webpages advised against specific interventions. Nineteen webpages (10%), while mentioning vitamins (six of them vitamin C), diet or probiotics, had a negative stance on the use of supplements. These “supplement-negative” webpages were mainly from professional (*n* = 6), news (*n* = 4), and non-profit (*n* = 3) websites. The reasons behind this negative stance on supplements are various and reasons given include: vitamins can be obtained from the diet; claims are unsubstantiated; they are not effective; they add useless cost; or they can cause side effects. A sample of the use of the word “supplement” in this context using corpus analysis is provided in Supplementary File 2.

Finally, it is important to note that none of the 185 webpages retrieved in our search had an anti-vaccine or vaccine-skeptical stance.

### Commercial Bias and Newsworthiness

We asked whether commercial websites, having a financial interest, preferentially mention specific “boosts,” that is whether some of these were mentioned by commercial webpages with a higher frequency than in all other webpages in the SERP. To do so, we looked at the ratio between the frequencies of a topic in commercial webpages and compared it with the frequency of the same topic in all other webpages. Likewise, to investigate whether some of the topic were more newsworthy, we compared the frequency of the topics in news websites compared to all other websites in the SERP.

**Figure 3:**
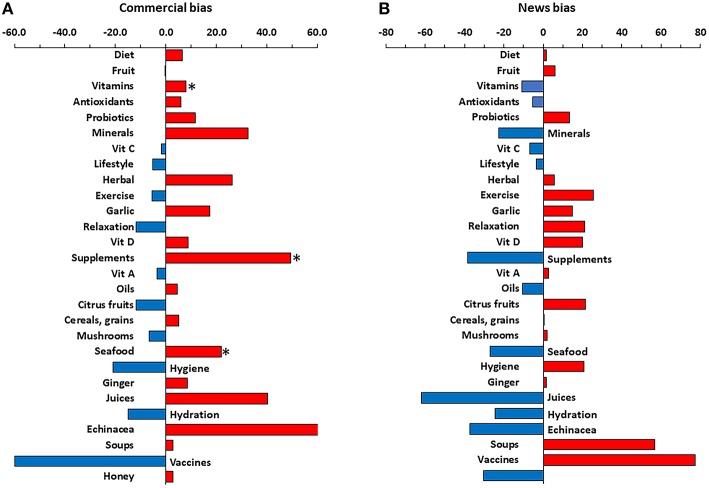
Differential representation of approaches to boost immunity by commercial webpages **(A)** and news webpages **(B)**. Data are calculated as: 100^*^(percent mention in commercial or news webpages – percent mention in the total 185 websites/percent mention in the total 185 websites. The expected value, in case of no bias should be zero. A value >0 denotes (blue) an overrepresentation (positive bias), a value <0 (red) an underrepresentation (negative bias). ^*^Significantly over-represented by a Fisher test corrected for multiple comparison by the method of Benjamini–Hochberg with a false discovery rate set at 5%. For significance testing, the frequency a boost is mentioned by commercial or news webpage is compared with its frequency in the remaining webpages in the SERP, rather than with that in the whole SERP, to avoid comparing two groups with a significant amount of overlapping data.

The analysis of commercial bias is shown in Figure 3A. Minerals, supplements, juices, and Echinacea were highly mentioned by commercial webpages (1.6, 2.0, 1.7, and 2.3 times more than the rest of the SERP, respectively). Conversely, vaccines were mentioned less frequently than in the rest of the SERP. The over-representation of “minerals” and “supplements” in commercial webpages was statistically significant (Fisher's test corrected for multiple comparisons using the Benjamini–Hochberg correction set at a false discovery rate of 5%).

In the same way, newsworthiness is shown in Figure 3B. Vaccines were mentioned 2.5-times more frequently by news webpages than the rest of the webpages, and so did “soups” (the word mostly occurred in “chicken soup”), although these differences were not statistically significant after correction for multiple comparisons.

Since many health news stories report on research studies, often relying heavily on press releases from Universities, their industrial sponsors and the academic journals they publish with (23), we counted the number of webpages mentioning the word “university.” This was done a posteriori by analyzing the corpora represented by the text of the “news” webpages downloaded automatically (see Materials and Methods) and compared with three other typologies (commercial, professional, and blog). Because not all websites allow access to robots, only a sample, although representative, of these webpages could be downloaded analyzed (68% of news webpages, 54% of commercial, 50% of professional, and 39% of blogs). The results showed that the word “university” was mentioned in 44% of news webpages compared to 19% of commercial, 25% of professional, and 14% of blogs. Full analysis of the concordance for the word “university” in news webpages shows that it was used in the context of reporting research results (Supplementary File 3). In some cases, this represented a reference to a statement by an academic professional, in other cases there was a reference to a scientific publication, although the publication was not always identified. For instance three news webpages (no. 15, 21, and 188 in the Supplementary File 1) mention a study from the University of Vienna reporting that daily consumption of yogurt is as effective in stimulating immunity as taking probiotic pills, but we could not find a reference to that paper. These results indicates that news outlets are key in relaying results of academic research to the public.

## Discussion

The present study highlights a large presence of commercial websites, representing 30% of the whole SERP. It is also important to consider the ranking given by Google. In fact, many studies indicate that people often visit only the first websites presented in a SERP. In terms of typology of websites, we observed that Google tends to rank commercial websites low, as none of them appears in the top 10 hits, giving more visibility to news websites. The low raking given to commercial websites is something that we observed previously with other search queries (15, 17). Of note, commercial webpages also ranked lowest in intrinsic (content-independent) HIQ criteria such as the JAMA score and the HONcode, which we also observed in other studies (15, 16) and may contribute to the lower ranking.

The content analysis focused on two aspects, the disease area and the approach suggested boosting immunity. Clearly, the focus of most webpages is on infections, particularly respiratory infections (influenza and common cold), in agreement with the scientific knowledge on the main role of the immune system as a defense against pathogens. This focus on infection is confirmed by a number of approaches described (hygiene, like washing your hands) that are more about preventing an infection by different means than boosting immunity.

In terms of “boosters,” most webpages mentioned dietary advice, fruit or vegetables, and vitamins, particularly vitamin C. It should be noted, however, that when vitamins taken as supplements are suggested, the webpage would also be tagged as “supplement.” Just 36% of webpages specifically mentioned supplements (including vitamin C, flavonoids, minerals, and other agents) to boost immunity. It seems therefore, that the main message from most sources of information is that a “healthy diet,” rich in fruit and therefore vitamins, is an immune booster. One comment is that in many cases the information provided is not answering the query “boosting immunity,” as a “boost” should be something that stimulate immunity above the normal level, but rather informs on how to avoid immunodeficiency due to, for instance, malnutrition.

The high frequency with which vitamin C is mentioned is in agreement with a US survey indicating that, of all dietary supplements, vitamin C is the only one for which the most commonly reported motivation for its use (in 15% of the subjects) is “to boost immune system, prevent cold” (24). “Strengthen the immune system” is also a major reason (54% of the respondents) for the use of dietary supplements in cancer survivors (25).

It is important to note that most of the boosters mentioned are not incorporated in any medical guidelines and represent information that is around CAM. This is also true for some of the nutritional advice given (e.g., garlic, mushrooms, ginger, soups), which is either based on low-level (preclinical) scientific evidence or on traditional medicine. It should be noted, however, that the marketing of supplements is not subject to the same level of evidence-based medicine required to market a medicinal product [discussed in (26)], as long as the claim is not addressing a specific disease. For instance, the European Food Standard Authority (EFSA) approved the claim that vitamin C “contributes to maintenance of the normal function of the immune system” (27).

The high frequency with which vitamin C is mentioned in the context of boosting immunity raises the question of how it became so popular in this field and in the public understanding of science (28). This does not seem to reflect clinical evidence (29) or research activity in terms of scientific publication. A search on PubMed for the term “immunity” on 31/1/2019 returned 453867 publications. Adding the term “vitamin C” returned just 306 papers (0.07%). In comparison, publications on immunity and “interferon” were 9%, immunity and “vaccine” 13.7%, immunity and “antioxidant” 2.1%, immunity and “diet” 1.5%, and immunity and “vitamin D” 0.29%. What is surprising is that vaccination, the only approved means of (literally/actively) boosting immunity against specific pathogens, ranks 27th, with only 12% of webpages mentioning it.

The highest commercial bias was around Echinacea and supplements in general. It is also not surprising, although worrisome, that these webpages, often oriented to a “natural” approach, only three out of 60 mention vaccines, although we could not identify any anti-vaccine or vaccine-skeptical webpage in the SERP. Of course natural therapies can be used as complementary, in addition to vaccination, or as an alternative to vaccination. Browne et al. suggested that vaccination compliance might be increased by appealing to features usually associated with CAM, e.g., “strengthening your natural resistance to disease” (13). Our study suggest that public health websites, educational websites, health portals, and professional organizations should specifically mention that vaccines “boost immunity” against infectious disease as otherwise their websites, and vaccines, would not be found after a search on this topic.

In conclusion, our study found that a search on boosting immunity returns a significant number (30%) of commercially-biased webpages promoting the use and sales of supplements. On the other hand, the ranking of webpages by Google gives more visibility to professional websites and news (4-times and 2-times more likely to rank in the top 10, respectively) while no commercial website was ranked high, none of them being in the top ten. In addition, none of the webpages in the search provided negative information about vaccines. Of course, while this study was performed of a sample of a reasonable size, this is the sample returned from a specific query, selected based on its popularity. Performing a more focused search on a specific supplement or disease may provide a different set of webpages. Furthermore, while the classification of most typologies was reliable, for health portals the inter-rater agreement was very poor and we could not draw any conclusion on this type of websites. Another important aspect is which language or localized version of Google is used, as a similar study on anti-vaccine website has shown major differences across different countries and languages (17). This implies that the sample uses is only representative of English language.

## Data Availability

All datasets generated for this study are included in the manuscript and the supplementary files.

## Author Contributions

PG designed the study. PG, AC, and AO collected and analyzed the data and wrote the manuscript.

### Conflict of Interest Statement

The authors declare that the research was conducted in the absence of any commercial or financial relationships that could be construed as a potential conflict of interest.

## Supplementary Material

Supplementary File 1 List of webpages analyzed and their classification.

Supplementary File 2 Concordance of the key word “supplement” is webpages recorded as having a negative stance of supplements.

Supplementary File 3 Concordance of the key word “University” in news webpages.
